# Laparoscopic-Assisted Anorectoplasty for Anorectal Malformation With Recto-Prostatic Urethral Fistula: A Case Report and Review of the Literature

**DOI:** 10.7759/cureus.49008

**Published:** 2023-11-18

**Authors:** Maria Florou, Chrysostomos Kepertis, Vassileios Mouravas, Kleanthis Anastasiadis, Ioannis Spyridakis

**Affiliations:** 1 2nd Department of Pediatric Surgery, Aristotle University of Thessaloniki, Papageorgiou General Hospital of Thessaloniki, Thessaloniki, GRC; 2 Department of Pediatric Surgery, General Hospital Papageorgiou, Thessaloniki, GRC

**Keywords:** pediatric surgery, urethral fistula, anorectoplasty, laparoscopic, anorectal malformation

## Abstract

Congenital recto-urethral fistula is the most common form of anorectal malformation found in boys. The final repair includes the ligation of the fistula and the anorectoplasty, and can be achieved either way: posterior sagitally or laparoscopically. We present a case of a term male infant diagnosed with anorectal malformation and recto-prostatic urethral fistula, that underwent a laparoscopic-assisted posterior sagittal anorectoplasty in our department.

## Introduction

Anorectal malformations include a variety of congenital anomalies, affecting boys and girls that involve congenital anomalies of the distal anus and rectum, as well as the urinary and genital tracts. Their incidence is approximately 1:5,000 live births [1]. The traditional classification by Wingspread, still in widespread use, divides these clinical entities into high, intermediate, and low anorectal anomalies, according to the relation of the distal rectum with the levator muscle and the pelvic floor [2]. A more practical classification followed, that describes the anatomic characteristics of the malformations. The recto-urethral fistula is the commonest form of anorectal malformations found in boys and is further divided into recto-prostatic type and recto-bulbular type of fistula [1]. Here we present a case of a laparoscopic-assisted anorectoplasty (LAARP) applied in a boy with high anorectal malformation. The procedure was performed for the first time in our surgical department. The patient recovered smoothly, and the results of this technique are closely observed in the long-term follow-up.

## Case presentation

**Figure 1:**
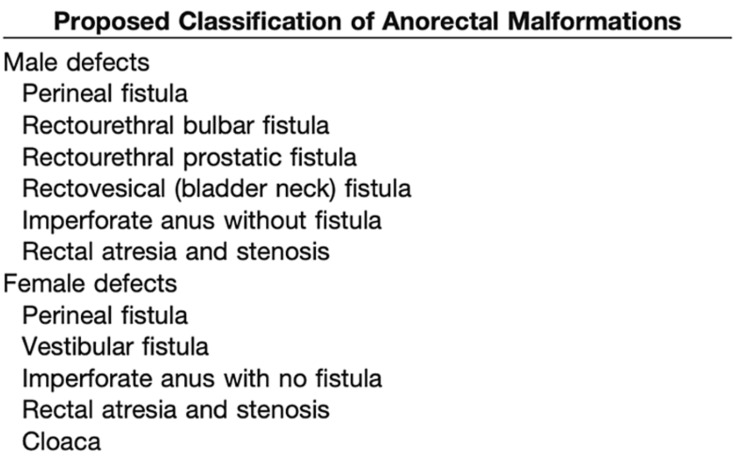
Current classification of anorectal malformations Suggested classification by Dr. Peña.

The patient was a male term infant, admitted to the neonatal intensive care unit of our hospital directly after birth, as an imperforate anus was discovered in the initial assessment. Physical examination after admission ascertained the anal atresia and a rectourethral fistula was suggested by abdominal ultrasonography (Figure 1) [3].

The genitalia were normal and there were not recognized no other congenital anomalies. On the second day of life, a divided colostomy of the sigmoid colon was applied in the left iliac fossa, in order to relieve the infant from the excessive abdominal distention. Postoperatively, the infant recovered well, gained weight as expected, and was discharged home until the scheduled anorectoplasty when the baby was one year old. A recto-prostatic urethral fistula was confirmed by a post-operative distal colostogram and a voiding urethrography.

**Figure 2:**
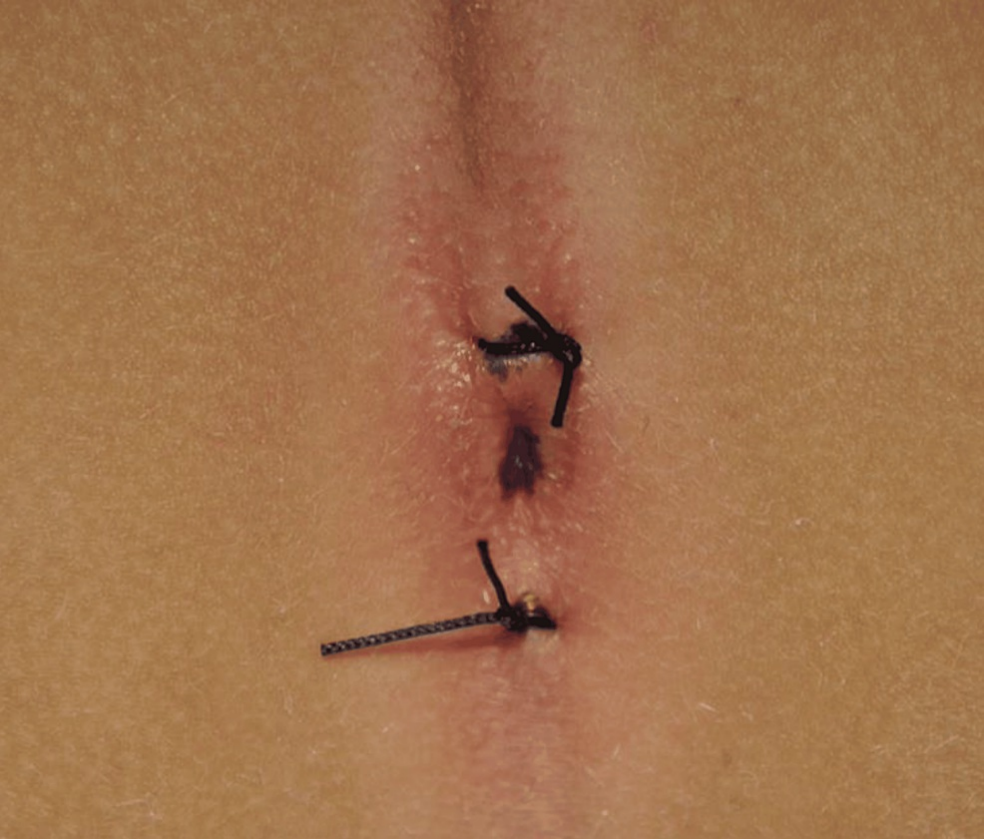
The placement of the anus was marked on the perineum pre-operatively.

The diagnosis of high anal atresia was established, and we decided to perform laparoscopic-assisted anorectoplasty. The placement of the anus was marked on the perineum pre-operatively after the application of transcutaneous electrostimulation (Figure 2).

**Figure 3:**
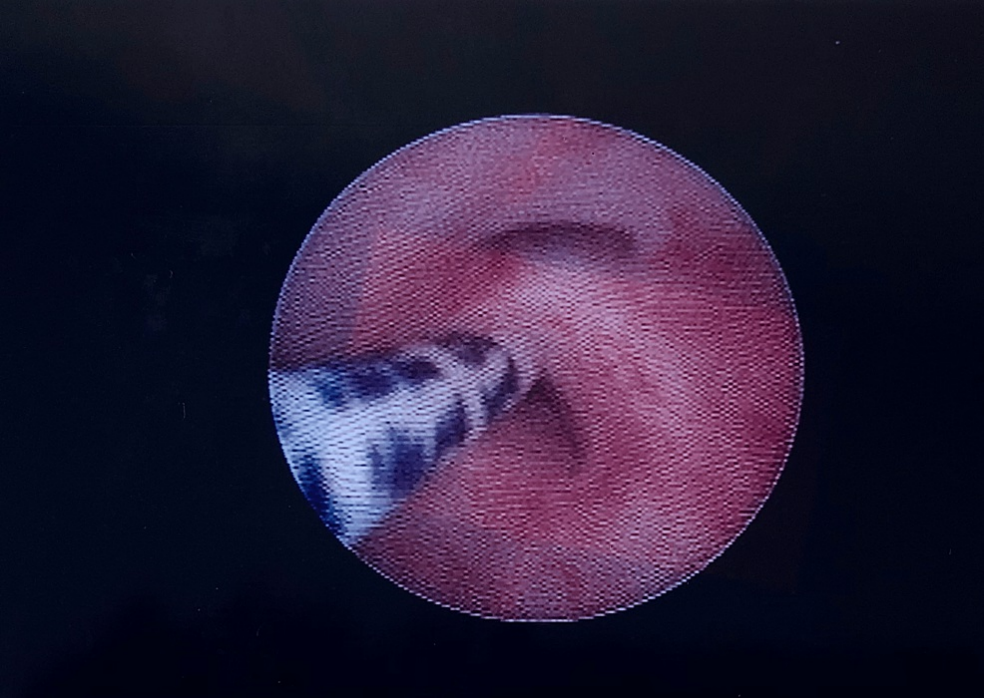
Cystoscopy detecting the recto-urethral fistula in the posterior wall of the prostatic urethra.

Cystoscopy was performed first, and the diagnosis of a recto-urethral fistula was confirmed by detecting the fistula in the posterior wall of the prostatic urethra (Figure 3).

**Figure 4:**
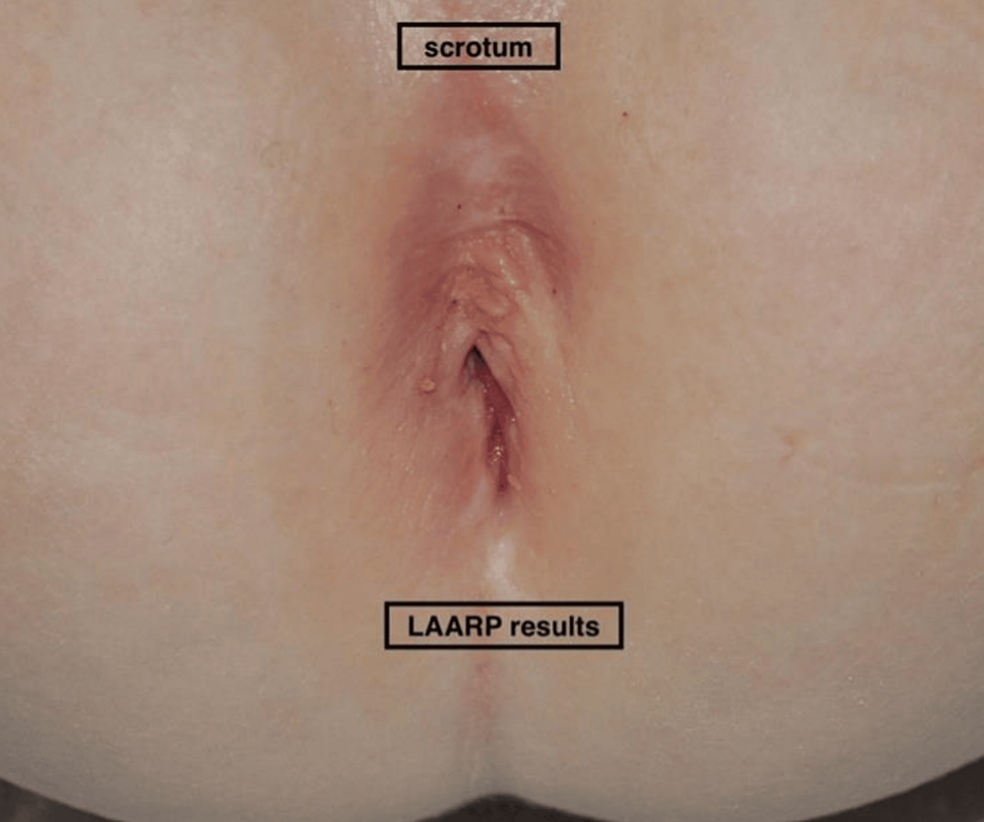
Cosmetic results of anorectoplasty seven years after the LAARP.

The applied technique involved inserting a 10-mm trocar through the umbilicus for the 30º laparoscope with the Hasson method, as well as a 3-mm trocar at the left iliac fossa, a 5-mm port and a 3-mm port at the right iliac fossa for the instruments. Rectal dissection began at the peritoneal reflection and continued on a rectal plane until the rectal blind pouch was pulled up to expose the fistula. The blunt dissection was made close to the rectal wall to minimize potential injury to anatomical structures, such as the ureters, the vas deferens, nerves, and vessels. The fistula to the urethra was identified, then was closed with a Polydioxanone (PDS) synthetic, absorbable, monofilament suture, size 5-0, and was sharply dissected to the most distal point. Transcutaneous electrostimulation was applied on the perineum and the area of maximal contraction was marked with silk sutures for the placement of the anus (Figure 3). Then a 10-mm vertical midline incision was made in the middle of this marked area and a tunnel through the perineal center of the sphincter complex was created by using artery forceps and a Veress needle. The anorectoplasty followed as suggested by Georgeson et al. [4]. More specifically, the tunnel was then dilated under laparoscopic vision using trocars gradually to 5 mm and then to 12 mm, just posterior to the urethra. The distal rectal pouch was pulled onto the perineum with Babcock forceps while the trocar was removed. The anastomosis between the rectum and the marked neoanus was completed with an interrupted 5-0 polydioxanone (PDS) suture. Post-operatively the boy received intravenous analgesics for four days and wide-broad spectrum antibiotics (cephalosporin second generation, metronidazole, and amikacin) for seven days. He recovered well and was discharged home. The closure of the sigmoidostomy followed four months later uneventfully. The patient presented good functional outcomes and fine cosmetic results, in the immediate post-operative period. The child gained weight and thrived as expected. There were no major complications, apart from a mucosal prolapse and a small posterior urethral diverticulum. The mucosal prolapse was found through clinical examination, two years after the anorectoplasty, and the patient was scheduled for a prompt fixation of the rectum. The urethral diverticulum was discovered in a planned urethrography, three years after the LAARP. Since then, the boy has not mentioned any related clinical symptoms, as urine incontinence or urine infection, the urine laboratory tests are normal, and the diverticulum remains the same size in the follow-up examinations: pelvic magnetic resonance imaging and voiding cysto-urethrography. The patient presents regularly at the surgical department in the arranged appointments and no problems are reported in the long-term follow-up (Figure 4).

It has been seven years since the LAARP procedure so far, the boy has good bowel control and mentions completely voluntary bowel movements, without any incidence of fecal incontinence, soiling, or serious constipation.

## Discussion

This was the first case of anorectal malformation treated with a laparoscopic approach in our department. The open surgical correction has been known to be the most effective management, but recently new approaches have been proposed. More specifically, two milestones can be recognized in the surgical management of anorectal malformations. The first was in 1980 with the introduction of the posterior sagittal approach by Alberto Peña. The posterior sagittal anorectoplasty (PSARP) enabled surgeons to fix the congenital defects under direct vision of the rectum, the genitourinary tract, and the muscle complex [3,5]. Since then, it has been considered the predominant technique in the management of anorectal malformations with good functional outcomes and rare severe complications [5]. The second milestone in the surgical repair of anorectal malformations was the introduction of the laparoscopic-assisted approach in 1998 by Willital [6]. The first LAARP for the repair of imperforate anus was applied in 2000 by Georgeson et al. [4]. The procedure combines minimal perineal dissection, preservation of the distal rectum, and precise placement of the rectum within the levator ani and external anal sphincter muscle complex. Laparoscopic surgery has been suggested to be also a safe approach for congenital anorectal anomalies with urinary fistula. LAARP technique has gained popularity since its introduction in 2000 and many studies followed that compared this technique to the gold standard PSARP method [3,4]. Regarding the operative time of each procedure, there are studies that report the longer time of the LAARP, while others mention the longer surgical time of the PSARP [7,8]. The literature data is inconclusive as the operative time depends on the complexity of the ARM, the patient comorbidities, and the skills of the different surgeons [9]. The cosmetic results, the wound infection incidence, the requirement of postoperative analgesia, and the consequent surgical stress, are in favor of the LAARP technique. The development of small-size instruments for the laparoscopic approach and the small 3-5 mm incisions are in favor of the LAARP technique, as on the contrary the extensive perineal dissection of the PSARP predisposes the patient to increased risk of wound infection and post-operative pain and stress [10]. The literature data on the functional clinical results, including the frequency of bowel movements, constipation, and soiling, either report no differences between the two procedures [11,12] or estimate better results of the LAARP approach [10,13]. On the other hand, the occurrence of rectal mucosal prolapse has been reported higher in LAARP treatment [7,14], as well as the incidence of posterior urethral diverticulum is more commonly seen in the follow-up of the laparoscopic management [15,16]. These two post-operative clinical entities are not considered major complications and were also noticed in the present case report. Last but not least the long-term follow-up of the two techniques on the voluntary bowel movements and the soiling presents similar rates for the two techniques and improved results for the constipation rates after the LAARP [14]. In overall, LAARP compared to the gold standard PSARP is less invasive and presents short-term and long-term outcomes similar if no better than PSARP [9,14].

## Conclusions

In conclusion, we performed laparoscopic-assisted anorectoplasty after a temporary divided colostomy, for a male imperforate anus with recto-bulbar urethral fistula, without any major problems. The treatment of children affected by anorectal malformations is still considered a challenge for the pediatric surgeon. When compared to the gold standard PSARP technique, both LAARP and PSARP can successfully treat anorectal malformations. More long-term, large studies with high-quality evidence are needed in the future to confirm the current literature data.
